# Advances in anesthesia technology are improving patient care, but many challenges remain

**DOI:** 10.1186/s12871-018-0504-x

**Published:** 2018-04-13

**Authors:** D. John Doyle, Ashraf Dahaba, Yannick LeManach

**Affiliations:** 1 Cleveland Clinic Lerner College of Medicine of Case Western Reserve University, Cleveland, Ohio USA; 2 Department of General Anesthesiology, Cleveland Clinic Abu Dhabi, Abu Dhabi, UAE, PO Box 112412, Abu Dhabi, UAE; 3 Priv.-Doz. Dr.med.university, Division of General Anaesthesiology, Emergency- and Intensive Care Medicine, Medical University of Graz, Graz, Austria; 4 Departments of Anesthesia & Health Research Methods, Evidence, and Impact, Michael DeGroote School of Medicine, Faculty of Health Sciences, McMaster University, 1280 Main Street West Hamilton, Hamilton, ON L8S 4L8 Canada; 5 Population Health Research Institute, David Braley Cardiac, Vascular and Stroke Research Institute, Perioperative Medicine and Surgical Research Unit, 237 Barton Street East, Hamilton, ON L8L 2X2 Canada

## Abstract

Although significant advances in clinical monitoring technology and clinical practice development have taken place in the last several decades, in this editorial we argue that much more still needs to be done. We begin by identifying many of the improvements in perioperative technology that have become available in recent years; these include electroencephalographic depth of anesthesia monitoring, bedside ultrasonography, advanced neuromuscular transmission monitoring systems, and other developments. We then discuss some of the perioperative technical challenges that remain to be satisfactorily addressed, such as products that incorporate poor software design or offer a confusing user interface. Finally we suggest that the journal support initiatives to help remedy this problem by publishing reports on the evaluation of medical equipment as a means to restore the link between clinical research and clinical end-users.

Advances during the last several decades have led to important improvements in clinical monitoring technology and clinical practice development, not only in patients undergoing surgery [1–6] or in patients being cared for in Intensive Care Units (ICUs) [7–9] but also in ambulatory patients [10, 11]. These developments have contributed to great improvements in patient safety [3, 5–7, 12–14]. In addition, anesthesiologists world-wide have developed standards for continuous real-time monitoring of hemodynamics, oxygenation, ventilation, neurological status, urine output, core temperature, degree of neuromuscular blockade, as well as other items, all of which have also contributed significantly to patient safety [15–17].

Several other innovative developments have also contributed to improving the quality of perioperative care. Checklists, proven to be particularly valuable in the aerospace industry, are now in common use in the operating room and elsewhere [18–22]. For example, in a landmark study by Haynes et al. [23], a surgical death rate of 1.5% before the introduction of a surgical checklist fell to 0.8% after, with an inpatient complication rate dropping from a baseline of 11% down to 7% after introduction of the checklist. New approaches to clinical airway management such as airway algorithms [24, 25], video laryngoscopy [26–31], extubation catheters [32–34] and advanced supraglottic airway devices [35–38] are also protecting patients from injury.

In the realm of perioperative cardiac monitoring, the use of conventional and 3D- echocardiography [39, 40] now allows for real-time monitoring of valvular function, ventricular filling, cardiac contractility and other hemodynamic parameters. Additionally, hand-held ultrasound machines are changing how bedside examinations are conducted [41–44] (Fig. 1). Clinical early warning algorithms, especially valuable in the perioperative setting to detect the early onset of clinical deterioration, have also proven to be effective in improving patient care [45–49].

**Fig. 1:**
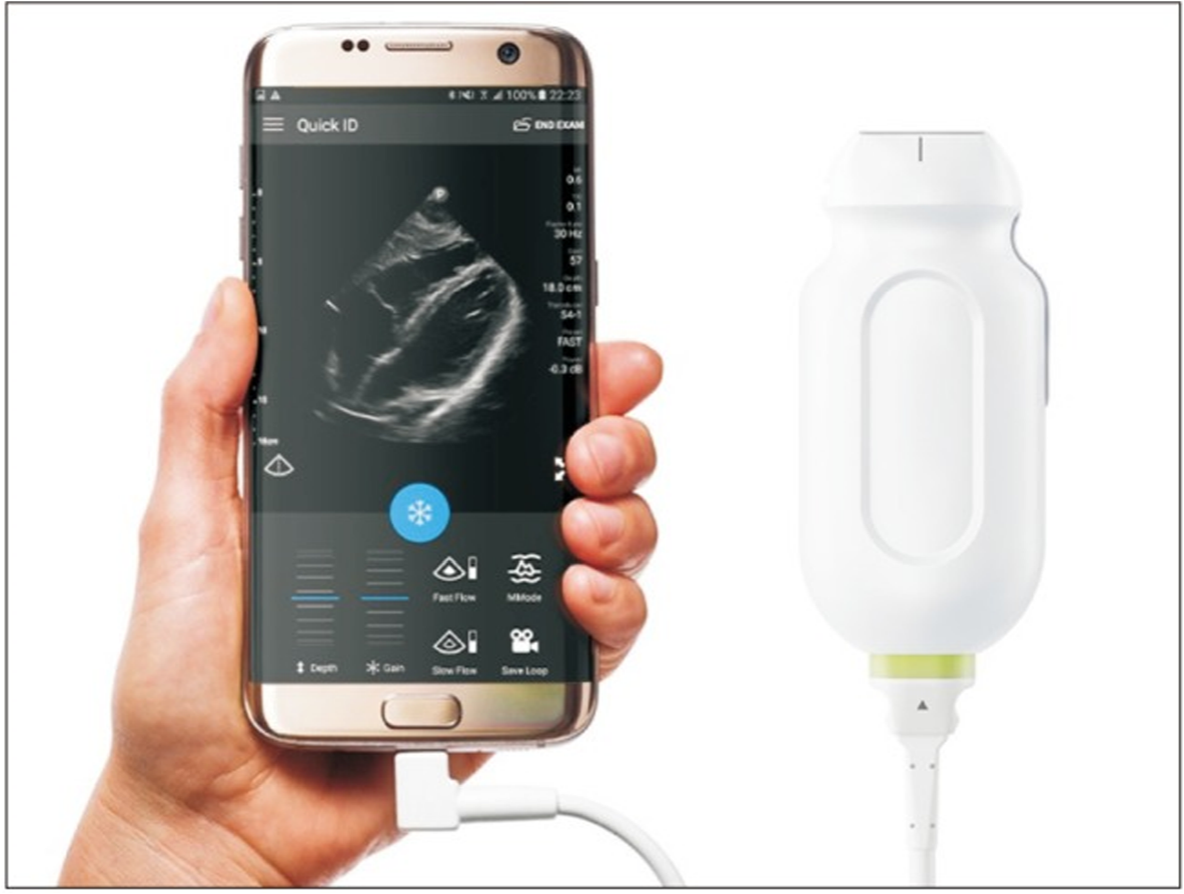
Ultrasound machines for applications such as echocardiography, regional anesthesia or central line placement have now evolved to the point that they can connected to a smartphone or tablet.Image from Michard F. Smartphones and e-tablets in perioperative medicine. Korean J Anesthesiol. 2017 Oct;70(5):493–499. doi: 10.4097/kjae.2017.70.5.493. PubMed PMID: 29046768; PubMed Central PMCID: PMC5645581*.* Image used under the terms of the Creative Commons Attribution Non-Commercial License, which permits unrestricted non-commercial use, distribution, and reproduction in any medium, provided the original work is properly cited

Another important development concerns the numerous so called “Depth of anesthesia monitors” such as the bispectral index (BIS) monitor. Historically, bispectral analysis is a standard high-order statistical analysis first used by oceanographers as a measure of time series to study nonlinearity in ocean waves [50]. This was further developed in the 1960s by geophysicist seismologists to study complex seismic waveforms [51]. The first EEG-derived monitor, the BIS (Medtronic, Dublin, Ireland) was introduced in 1994 as a monitor of the effects of certain anesthetic/hypnotic agents [52]. To date the complete details of the BIS algorithm have not been published. Scientifically speaking, all that we really know is that the BIS monitor is merely a “black box” headset and the BIS value reflects a “head-related” biosignal that correlates with changes in the biophase effect-site concentrations of certain hypnotic /sedative drugs and thus cannot be considered a “true” reflection of the depth of anesthesia. In other words, the BIS index is a measure of certain drugs’ effect and is not a true reflection of the EEG signal nor an independent measure of brain function [53]. In the early years all attempts of the manufacturer for the Food and Drug Administration (FDA) to license the BIS as an “independent uniform depth of anesthesia” monitor failed. For instance, a BIS value of 57 for 1 MAC halothane is significantly higher than the BIS value of 32 for an equipotent 1 MAC sevoflurane [54], and significantly higher than a BIS value of 33 for an equipotent 1 MAC isoflurane [55]. While ketamine provides adequate anesthesia, ketamine administration was reported to paradoxically increase the BIS from 44 to 59 [56].

What is the bispectral index then? The BIS algorithms were derived from EEG changes with incremental doses of certain hypnotic drug(s); isoflurane or propofol while measuring 3 descriptors in volunteers. The BIS index is the weighted sum of 3 sub-parameters; *Relative BetaRatio* most influential during light anesthesia, a frequency–domain feature is the EEG spectral power log (P_30–47 Hz_ /P_11–20 Hz_), *SynchFastSlow* predominates during surgical levels of hypnosis, a bispectral–domain feature, is the bispectral power wave band log (B_0.5–47 Hz_ /B_40–47 Hz_) and *Burst Suppression* that detects very deep anesthesia, a time–domain feature combining two separate algorithms: Burst Suppression Ratio that quantifies the extent of isoelectrical silence, and QUAZI suppression index that detects Burst Suppression superimposed on wandering low baseline voltage [57]. None of these disparate descriptors is particular per se; as each has a specific range of influence where they perform best. The BIS analysis uses a proprietary algorithm that allows the 3 different descriptors to sequentially dominate as the EEG changes its character with increasing anesthetics’ concentrations. It thus transforms the nonlinear stages of the anesthetic drug effect relative contributions on the EEG into an easy-to-use dimensionless number ranging from 100 (fully awake) to zero (isoelectric EEG) [57].

Obviously, in many instances BIS changes do not truly reflect changing anesthetics’ concentrations, as BIS indices would reflect other unrelated EEG events of certain conditions exerting their own EEG effect. Because the BIS is an EEG derived parameter hence anything that would change the EEG would subsequently change the BIS. There is a body of literature of EEG changes of conditions like hypothermia, hypoglycemia, hypovolemia, hypotension, hepatic encephalopathy or physiological sleep with the same conditions consequently changing the BIS to the same extent [58].

In the realm of the ever-changing landscape of neuromuscular blockade monitors, older designs are frequently replaced with new devices that are often promoted as technically superior by the manufacturers. Conventional mechanomyography (MMG) is regarded by the Stockholm revision consensus conference [59] as the gold standard for precise quantification of neuromuscular block, as it quantifies the exact force displacement isometric muscle contraction of a preload-restrained thumb in response to electric stimulation at the ulnar nerve [33]. The main obstacle facing its wide clinical use is that the equipment takes time to set up and requires rigid support of the arm.

Over the years, we have seen numerous stand-alone or modular-integrated neuromuscular monitoring devices that quantify the neuromuscular function based on physiological phenomena other than force measurement. The kinemyographic (KMG) device known as ParaGraph [60] (Vital Signs, Totowa, NJ) is no longer available for routine clinical use as the manufacturer has been acquired by CareFusion in 2014 although the neuromuscular transmission module (E-NMT) in the AS/5TM anaesthesia monitor (GE, Helsinki, Finland) [61] is still available. Both quantify the signal generated from thumb adduction via deformation of a piezoelectric film sensor in response to electric stimulation of the ulnar nerve. E-NMT has an additional electromyographic (EMG) transducer that quantifies the evoked compound action potential generated at the thenar eminence.

Another attractive class of devices are acceleromyographic (AMG) monitors. The first commercially available product, TOF-GUARDTM (Organon Teknika, Oss, Netherlands) [62]. now discontinued, has been replaced by a simpler device known as TOF-WatchTM (MIPM, Mammendorf, Germany) [63]. Both measure the acceleration using a piezoelectric sensor attached to a freely moving thumb (“piezo” from the Greek word meaning pressure). Note that according to Newton’s second law: force = mass x acceleration, acceleration is directly proportional to force when mass is constant, so that instead of measuring the evoked force, thumb acceleration can be measured instead. A major impediment of this type of monitoring is the fact that the piezoelectric sensor may not always be “properly aligned” to the optimal plane of the thumb movement. A comprehensive systematic review of acceleromyography by Claudius and Viby-Mogensen described many of the methodological problems facing the technology based on evidence based data of 43 publications [63].

With the manufacturer official announcement of the discontinuance of all the TOF Watch monitors series effective June 2016, this development gave way to a new wave of neuromuscular monitoring devices, namely a novel generation of so-called Tri-axial acceleromyographs. Nowadays the only commercially available acceleromyographs all belong to the new Tri-axial based generation; namely the Stimpod NMS 450 (Xavant, Silverton, Pretoria, South Africa), the TOFscan (Dräger, Lübeck, Germany) and the new modular neuromuscular transducer NMT (Mindray, Shenzhen, China).

The above successes notwithstanding, many vital challenges remain to be addressed by the anesthesia technology community. One of these challenges includes reducing the time interval needed to troubleshoot a malfunctioning electrocardiogram, capnograph, pulse oximeter or some other patient monitor prior to starting an anesthesia case. Another challenge is in the realm of alarms [2, 4, 64]; who has not been irritated when the source of a monitor alarm is completely unapparent or when an asystole alarm occurs despite both a good arterial blood pressure waveform and a high-quality pulse oximeter tracing being present. Such difficulties divert attention from direct patient monitoring as mental effort is expended to address some technical problem. Problems related to poor software design or careless user interface designs have also led to patient harm [65–68].

In view of these concerns, we would like to propose that the journal support initiatives by publishing reports on the evaluation of anesthesia and perioperative equipment [69]. These reports might be made in a manner not dissimilar to information provided by web sites like eopinions.com as well as in specialized magazines like Consumer Reports (which provides evaluations of products such as household appliances) or in reports provided by a number of Personal Computer magazines (focusing on software and hardware products).

We envision two general forms of report. The first kind of report would be an informal “first impressions” description of newly available equipment. These reports – or user’s opinions -would frequently make observations concerning ergonomics and equipment usability. Extensive and definite evaluation would not be the primary objective. While necessarily subjective, this information would be valuable to individuals seeking to acquire new equipment. Further, individual feedbacks about a newly available equipment will be of major interest to coordinate clinical evaluations based on structured evaluation protocols in a collaborative effort regrouping clinicians familiar with the equipment.

A second, more formal, kind of report would be supported based on the usual scientific publication presentation. This kind of report would be based on rigorous, reproducible testing methods like those methods used by ECRI (ecri.org) and other testing agencies to produce detailed, formal, laboratory-based assessments. Additionally, these reports would include clinical evaluations based on broadly discussed evaluation protocols including modern statistical methods. Because of the nature of the devices under evaluation, research protocols tend to vary in objectives, in design and in quality. Collaborative efforts based on a single well-structured design is sometimes the key to obtain timely clinical evaluations of a new device.

The combination of rapid feedback and qualitative structured evaluations of new equipment will allow anesthesia community to focus on the device of interest in a timely matter. Immediate feedback has the potential to improve the design of new devices, and collaborative evaluation effort is usually the fastest way to obtain sufficient data to reliably draw conclusions about the clinical and economical value of a new device.

We believe the journal can play an important role in this initiative. With a more interactive, a more collaborative, and a more international approach, such an initiative would help restore the link between clinical research and clinical end-users. Further, by quickly producing high-quality, clinically relevant evaluations, we believe this initiative could have a long-lasting impact on medical device design as well as ultimately on patient safety.

## Abbreviations

AMG: Acceleromyogram acceleromyographic
BIS: Bispectral index
ECRI: Emergency care research institute
EEG: Electroencephalogram
ICU: Intensive care unit
KMG: Kinemyogram / kinemyographic
NMT: Neuromuscular transmission
TOF: Train of four
