# Correction to: Advances in anesthesia technology are improving patient care, but many challenges remain

**DOI:** 10.1186/s12871-018-0517-5

**Published:** 2018-05-30

**Authors:** D. John Doyle, Ashraf A. Dahaba, Yannick LeManach

**Affiliations:** 1 Cleveland Clinic Lerner College of Medicine of Case Western Reserve University, Cleveland, Ohio USA; 2 Department of General Anesthesiology, Cleveland Clinic Abu Dhabi, Abu Dhabi, UAE, PO Box 112412, Abu Dhabi, UAE; 3 Priv.-Doz. Dr.med.university, Division of General Anaesthesiology, Emergency- and Intensive Care Medicine, Medical University of Graz, Graz, Austria; 4 Departments of Anesthesia and Health Research Methods, Evidence, and Impact, Michael DeGroote School of Medicine, Faculty of Health Sciences, McMaster University, 1280 Main Street West Hamilton, Hamilton, ON L8S 4L8 Canada; 5 Population Health Research Institute, David Braley Cardiac, Vascular and Stroke Research Institute, Perioperative Medicine and Surgical Research Unit, 237 Barton Street East, Hamilton, ON L8L 2X2 Canada

## Correction

Unfortunately, after publication of this article [1], it was noticed that the name of Ashraf A. Dahaba is incorrectly displayed as Ashraf Dahaba. The full, corrected author list can be seen here.
